# To be or not to be

**DOI:** 10.7554/eLife.38967

**Published:** 2018-07-17

**Authors:** Zhe Zhang, Elisabeth Hessmann

**Affiliations:** Department of Gastroenterology and Gastrointestinal OncologyUniversity Medical Center GoettingenGottingenGermany

**Keywords:** pancreatic cancer, SWI/SNF, acinar cell, Arid1a, Kras, chromatin remodeling protein, Mouse

## Abstract

Chromatin remodeling processes can drive acinar cell fate decisions.

**Related research article** Livshits G, Alonso-Curbelo D, Morris JP, Koche R, Saborowski M, Wilkinson JE, Lowe SW. 2018. Arid1a restrains Kras-dependent changes in acinar cell identity. *eLife* **7**:e35216. doi: 10.7554/eLife.35216

Every single cell in the body contains the same genetic information. However, different types of cells activate distinct sets of genes at different times and locations, which allows them to carry out their precise roles. How do cells achieve this?

The answer lies in a specific structure called chromatin, which is formed of nucleosome units, in which a defined amount of DNA is wrapped around a core of histone proteins. To activate a specific gene, the chromatin first needs to be remodeled to provide access to the transcription machinery (Strahl and Allis, 2000). This process is firmly controlled by chromatin remodeling proteins, which disrupt the tight contact between DNA and histones, and mobilize the nucleosomes to reveal the ‘hidden’ genes (Owen-Hughes, 2003; Bossen et al., 2015).

Failures in the chromatin remodeling machinery can severely hamper the function of a cell, or worse, foster malignant transformations that can lead to cancer (Feinberg et al., 2016; Plass et al., 2013; Wilson and Roberts, 2011). This affects in particular the subunits of the chromatin remodeling complex called SWI/SNF (Kadoch et al., 2013). So far, it has remained unclear how mutations within this structure can lead to the development of tumors. Now, in eLife, Scott Lowe and colleagues from the Memorial Sloan Kettering Cancer Center, the Hannover Medical School and the University of Michigan – including Geulah Livshits as first author – report how a subunit of SWI/SNF, called Arid1a, is involved in the development of pancreatic cancer (Livshits et al., 2018).

Pancreatic cancer is one of the most aggressive types of cancer, with a five-year survival rate of less than 8% (Siegel et al., 2016). Around a quarter of pancreatic cancers contain mutations in the SWI/SNF complex, which are commonly accompanied by mutations in a gene called *Kras* (Hingorani et al., 2003). Now, Livshits et al. elegantly introduce the subunit Arid1a as a pivotal player in directing the fate of acinar cells – the cells that produce digestive enzymes to help break down food – in the presence of the cancer-causing or oncogenic *Kras*.

The researchers engineered a mouse model with a *Kras* mutation that allowed them to turn off Arid1a specifically in the acinar cells by feeding the mice antibiotics. When comparing the pancreas of adult mice with either a *Kras* mutation only, with deactivated Arid1a only, or with both a *Kras* mutation and deactivated Arid1a, the results revealed that the consequences of removing Arid1a were determined by the *Kras* mutation status and the time point of Arid1a depletion in relation to oncogenic activation of *Kras* (Figure 1).

**Figure 1.**
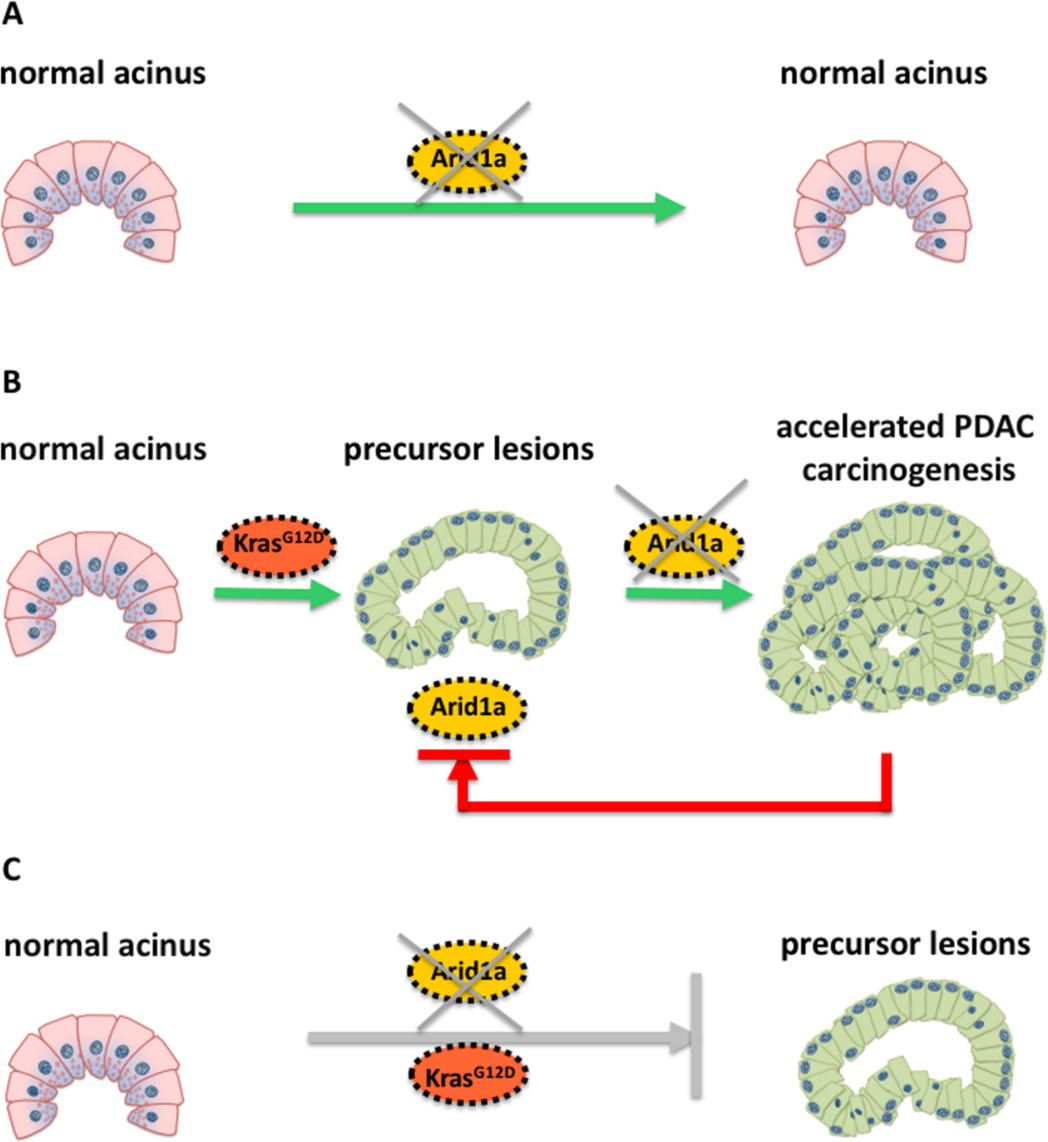
Schematic illustration of the molecular changes that can alter the identity of acinar cells in the pancreas. Livshits et al. show that a subunit of the chromatin remodeling protein SWI/SNF, called Arid1a, could contribute to the development of pancreatic cancer. (**A**) Mice without a *Kras* mutation and without Arid1a (yellow) did not show any cancer symptoms, suggesting that a lack of Arid1a alone cannot drive the reprogramming of acinar cells (pink). (**B**) Inactivating Arid1a in the context of a preexisting *Kras* mutation irreversibly boosts the development of pancreatic cancer (PDAC). (**C**) Removing Arid1a at the same time as activating the *Kras* mutation (Kras^G12D^, red) does not accelerate the formation of precursor lesions that could lead to pancreatic cancer.

Mice without a *Kras* mutation and without Arid1a did not show any precancerous lesions in their cells, suggesting that the SWI/SNF protein may be dispensable for maintaining the architecture of acinar cells in the absence of oncogenic *Kra*s (Figure 1A). However, inactivating Arid1a in the context of a preexisting *Kras* mutation significantly accelerated the symptoms of the mice within two weeks: the acinar cells of these mice started to transform into a different phenotype and stopped producing digestive enzymes – instead, they started making other proteins, such as mucins, which are typically found in precancerous or cancerous cells in the pancreas (Figure 1B). Most importantly, these symptoms remained irreversible, even when the antibiotics were removed.

Removing Arid1a in mice embryos at the same time as activating the *Kras* mutation did not have the same effect (Figure 1C): as the Kras mutation alone eventually leads to formation of pancreatic cancer. The thorough histological and molecular analyses of Livshits et al. demonstrate that a lack of Arid1a can increase the sensitivity of acinar cells to oncogenic signals, leading to the formation of pancreatic cancer. Nevertheless, Arid1a’s contribution to pancreatic carcinogenesis strongly depends on the molecular (mutated *Kras*) and temporal context (when the *Kras* mutation occurs).

Due to Arid1a’s ability to influence cell fate, a loss of Arid1a combined with a *Kras* mutation may therefore severely alter the composition of chromatin. Indeed, when Livshits et al. looked at the chromatin organization, it showed that mice without Arid1a had an abnormal chromatin structure, with genes encoding digestive enzymes being less accessible than in mice with normal Arid1a levels.

Together, the data by Livshits et al. characterize Arid1a as a critical but context-dependent gate keeper of acinar cell fate and pancreatic carcinogenesis. Since chromatin regulatory proteins control reversible processes, they represent promising targets for new therapeutic approaches in cancer treatment. Hence, disentangling the interdependence of the chromatin regulatory protein and context-defining molecular changes in the development of pancreatic cancer and other malignancies, constitutes a difficult but crucial challenge of future studies in the field.
